# Methionine and its derivatives in dairy cow nutrition: implications for intestinal barrier function, periparturient performance, and metabolic health

**DOI:** 10.3389/fvets.2025.1664853

**Published:** 2025-09-26

**Authors:** Wenying Huo, Yiyu Lin, Cailing Wang, Hongyu Deng

**Affiliations:** ^1^College of Animal Science and Technology, Henan University of Animal Husbandry and Economy, Zhengzhou, China; ^2^College of Animal Science and Technology, Qingdao Agricultural University, Qingdao, China

**Keywords:** methionine, gastrointestinal health, reproductive performance, dairy cows, embryonic development

## Abstract

The increasing demand for high-quality protein products has driven substantial progress in dairy cow nutrition, with a focus on optimizing amino acid supply to enhance productivity and health. Methionine (Met), a key essential amino acid, plays multifaceted roles in supporting growth, lactation, and reproduction, as well as maintaining metabolic and immune homeostasis in ruminants. Recent evidence highlights the regulatory potential of dietary Met—particularly in its rumen-protected form (RPM)—on intestinal integrity and systemic metabolic function in dairy cows. This review provides a comprehensive synthesis of the molecular properties and functional roles of Met and its derivatives in ruminant physiology. We evaluate current research on Met’s influence on the microbial, chemical, mechanical, and immunological components of the intestinal barrier, as well as its effects on milk synthesis, reproductive performance, and metabolic modulation during the periparturient period. While the benefits of Met supplementation are well-recognized, critical knowledge gaps remain regarding its mechanisms of action, interactions with gut microbiota, optimal dosing strategies, individual variability in response, and long-term outcomes across lactations. To address these gaps, future studies should adopt integrative multi-omics and microbiota-metabolite profiling approaches, develop precision nutrition models, and explore synergistic interactions with other nutrients. Advancing our understanding of Met’s roles in dairy cow nutrition will support the development of targeted supplementation strategies aimed at improving gastrointestinal health, reproductive efficiency, and overall productivity in commercial dairy systems.

## 1 Introduction

Amino acids (AAs), the fundamental monomeric units of proteins, are indispensable components in meeting the nutritional requirements of dairy cattle. AAs are categorized into essential amino acids (EAAs), which cannot be synthesized endogenously, and non-essential amino acids (NEAAs), which can be synthesized *de novo* within the animal (1). Among the EAAs, methionine (Met) plays a pivotal role in maintaining gastrointestinal integrity, modulating nutrient metabolism, and supporting productive and reproductive performance in dairy cows. As such, Met constitutes a critical determinant of bovine health and nutritional adequacy. Met exists in two stereoisomeric forms—L-Met and D-Met—with only the L-isomer being biologically active in protein synthesis (2). Met is derived from multiple sources, and natural L-Met occurs in various plant- and animal-derived feed ingredients, whereas synthetic DL-Met is industrially produced through the chemical reaction of methyl mercaptan, acrolein, and hydrogen cyanide. In commercial applications, DL-Met is often converted into N-acetyl-DL-Met, which is subsequently subjected to enzymatic resolution via L-aminoacylase to yield enantiomerically pure L-Met. Additionally, L-Met can be produced through microbial fermentation or enzymatic biotransformation of suitable precursors (2, 3). However, due to the complexity of microbial fermentation and its susceptibility to feedback inhibition, chemically synthesized Met remains the predominant form used in commercial ruminant feeds.

To enhance its bioavailability and prevent ruminal degradation, various rumen-protected Met (RPM) formulations have been developed. These include lipid- or polymer-coated DL-Met products, such as those encapsulated with ethylene–vinyl acetate (EVA), which offer high ruminal bypass efficiency. Methionine hydroxy analogs (MHAs), primarily in the form of 2-hydroxy-4-(methylthio) butanoic acid, exhibit substantial ruminal stability and are commonly incorporated into low-cost feeding programs. Furthermore, Met-based chelated mineral complexes, such as zinc methionine (Zn-Met), enable the simultaneous delivery of amino acids and trace elements with enhanced bioavailability, reduced antagonism, and improved metabolic efficiency. The expanding diversity of Met formulations reflects its critical role in optimizing health, metabolism, and production in modern dairy systems (3).

## 2 Materials and methods

### 2.1 Literature retrieval strategy

A systematic literature search was conducted across three English-language databases—Web of Science, PubMed, and X-MOL covering studies published between 1966 and 2025. The search included a combination of subject terms and free-text keywords, covering all available publications up to June 30, 2025. The primary subject terms used included “methionine,” “gastrointestinal health,” dairy cows,” “reproductive performance,” and “milk production.” The comprehensive search strategy incorporated the following Boolean logic: (“methionine “) AND (“microbial regulation “OR “short chain fatty acid” OR “tight junction” OR “immune regulation”) AND (“dairy cows”) AND (“reproduction performance” OR “lactation performance” OR “milk protein” OR “milk yield”).

### 2.2 Inclusion and exclusion criteria

Inclusion Criteria:(1) Studies where methionine is the primary additive; (2) Studies involving dairy cows as experimental subjects; (3) Studies investigating the methionine metabolism or its effects on dairy cows.

Exclusion Criteria: (1) Non-original research articles (e.g., reviews, commentaries); (2) Duplicate publications; (3) Studies for which the full text could not be obtained; (4) Articles not aligned with the primary research objectives of this review.

### 2.3 Literature screening and data extraction

All retrieved records were imported into Zotero 7.0 for reference management. Duplicate entries were removed, followed by a preliminary screening of titles and abstracts to assess relevance. Subsequently, full-text screening was performed to determine eligibility based on the inclusion criteria. Additionally, reference lists of the included articles were manually searched to identify further relevant studies. All processes of literature searching, screening, and data extraction were independently conducted by the first author.

### 2.4 Results

A total of 508 articles were identified through the initial search. After removing 118 duplicates, 390 articles remained for screening. Based on the inclusion and exclusion criteria, 110 studies were ultimately included in this review. Among these, methionine was predominantly administered through feed, with intervention periods lasting more than two weeks.

## 3 Structure and biochemical properties of met

Met is a sulfur-containing essential amino acid that serves as a critical determinant of protein conformation, cellular metabolism, and redox homeostasis. As the canonical initiating amino acid for eukaryotic protein synthesis, Met also contributes substantially to the hydrophobic architecture of proteins due to its nonpolar side chain –CH₂–CH₂–S–CH₃, which confers pronounced hydrophobicity and facilitates stable integration within the hydrophobic cores of tertiary and quaternary protein structures (4). The sulfur atom within this thioether moiety is chemically labile and readily undergoes oxidation to yield methionine sulfoxide (MetO), thereby introducing a polar functional group that alters the physicochemical properties of the molecule (5).

The oxidation of Met to MetO and its subsequent reduction back to Met constitute a reversible redox cycle with significant biological implications. This process exhibits stereospecificity and is catalyzed by distinct enzymatic systems. Methionine sulfoxide reductase A (MsrA), a bifunctional enzyme, specifically reduces the S-epimer (Met-S-O) of methionine sulfoxide, whereas the monooxygenase Mical catalyzes the oxidation of Met to the R-epimer (Met-R-O) (6). The existence of parallel enzymatic pathways for these stereoisomeric forms enables Met to act as a dynamic redox sensor and regulator within the broader context of oxidative stress and cellular signaling. Beyond its structural and redox functions, Met exerts pleiotropic effects on physiological processes relevant to dairy production. Evidence from *in vitro* studies demonstrates that Met enhances the synthesis of milk protein and lipid, as well as the proliferation of bovine mammary epithelial cells (BMECs), through activation of the SNAT2-PI3K signaling cascade (7). *In vivo*, periparturient supplementation with rumen-protected Met has been associated with significant improvements in milk yield, milk protein content, and milk fat concentration (8, 9). Furthermore, Met serves as a key precursor for the biosynthesis of critical biomolecules such as reduced GSH and taurine, both of which are integral to antioxidant defense and osmotic regulation (10). Additionally, Met functions as a primary methyl group donor in one-carbon metabolism, a pathway central to nucleotide biosynthesis, epigenetic regulation, and transmethylation reactions (11). Collectively, these multifaceted biochemical properties underscore Met’s indispensable role in optimizing the metabolic, antioxidative, and productive capacities of dairy cows.

## 4 Effects of met on gastrointestinal health in dairy cows

The gastrointestinal tract plays a fundamental role in nutrient absorption, immune surveillance, and host defense. Central to these functions is the integrity of the intestinal barrier, which acts as a multifaceted defense system against luminal antigens and pathogenic microorganisms. This barrier operates as a selectively permeable interface that facilitates the absorption of nutrients while restricting the translocation of pathogens and toxins, thereby preserving mucosal and systemic homeostasis. Structurally, the intestinal barrier comprises four interrelated components: the microbial barrier, chemical barrier, mechanical barrier, and immunological barrier—each contributing to the maintenance of intestinal integrity and function (12). The microbial barrier consists of commensal, symbiotic, and probiotic microorganisms that competitively exclude pathogens and produce antimicrobial compounds such as bacteriocins and short-chain fatty acids (SCFAs), which suppress the proliferation of pathogenic taxa. The chemical barrier, comprising SCFAs, mucins, and antimicrobial peptides (e.g., cathelicidins), contributes to the modulation of local immune responses, epithelial renewal, and pH homeostasis (12). The mucus layer, composed primarily of highly glycosylated mucin proteins secreted by goblet cells, serves as the first line of defense by physically separating the luminal microbiota from the epithelial surface. Dysregulation of mucin biosynthesis or structure has been implicated in the pathogenesis of various gastrointestinal diseases, including colorectal carcinoma and ulcerative colitis (13, 14). The mechanical barrier includes epithelial cells and tight junctions that function cooperatively to prevent pathogen infiltration. The epithelial barrier, formed by a monolayer of intestinal epithelial cells connected by tight junction (TJ) proteins, functions as a dynamic physical and biochemical interface that regulates paracellular permeability and immune signaling (15). Lastly, the immunological barrier is composed of gut-associated lymphoid tissue (GALT), intraepithelial lymphocytes, and various antigen-presenting cells, which collectively orchestrate innate and adaptive immune responses to luminal antigens and maintain immunological tolerance. Perturbations in any of these barrier systems compromise intestinal integrity and have been mechanistically linked to a wide spectrum of pathological conditions, including inflammatory bowel disease, metabolic syndromes and neuroimmune disorders (16–18). Met has emerged as a critical modulator of gut health by promoting mucosal integrity, supporting epithelial cell turnover, and regulating local immune function. Through these mechanisms, Met contributes to the preservation of intestinal barrier function and the prevention of barrier dysfunction-associated diseases.

### 4.1 Modulation of the microbial barrier

The gastrointestinal microbiota represents a metabolically active and immunologically engaged community that is essential for host digestion, immune regulation, and colonization resistance. In dairy cows, the microbiota plays a particularly critical role in fermentative digestion and nutrient extraction from fibrous feeds. Met, owing to its metabolic versatility and regulatory functions, has been shown to influence the structural composition and functional capacity of the gut microbiome, thereby contributing to microbial homeostasis and host resilience. During the periparturient period—characterized by profound physiological, metabolic, and immunological shifts—dietary interventions targeting gut microbial ecology are of particular importance. Supplementation with RPM has gained global attention for its ability to enhance metabolic efficiency and mitigate periparturient stress in high-producing dairy cows (19–22). Emerging evidence indicates that approximately 10% of supplemented Met escapes rumen protection and becomes available for microbial metabolism in the rumen ecosystem (23). This microbial utilization of Met has been associated with shifts in microbial community structure, including increased relative abundance of beneficial taxa such as Lactobacillus, Ruminococcus, and Faecalibacterium. These alterations suggest that RPM exerts prebiotic-like effects, selectively promoting the proliferation of commensal and probiotic microorganisms that contribute to improved gut health, enhanced short-chain fatty acid production, and competitive exclusion of opportunistic pathogens (23). As shown in Table 1, RPM supplementation during the transition period significantly modulates microbial diversity and ecological stability, which may translate into improved nutrient absorption, strengthened barrier integrity, and attenuated systemic inflammation. These findings underscore the potential of Met not only as a limiting amino acid for protein synthesis but also as a strategic modulator of the gut microbiota–host interface. Of note, the observed differences in the prevalence of certain bacterial taxa may reflect both the type of diet provided and the extent to which methionine escapes rumen degradation. While RPM can influence microbial populations by altering amino acid availability in the small intestine, other factors such as dietary composition, feeding management, and interactions with other nutrients also play important roles in shaping the rumen microbiota. Therefore, observed microbial shifts likely result from a combination of methionine supplementation and broader dietary and environmental influences.

**Table 1:** Summary of studies on the effect of met supplementation on the abundance of gastrointestinal microbes in dairy cows.

Supplementation level and duration	Observed abundance improvement	Reference
RPM 0.08% of Dry Matter Intake (DMI) (from 23 days prepartum to 30 days postpartum)	↑*Fibrobacter succinogenes;* ↓*Selenomonas ruminantium*	(23)
455 mg Zn-Met/d (2 weeks)	↑*Veillonellaceae,* ↑*Akkermansia muciniphila,* ↑*Bifidobacterium adolescentis;* ↓*Acinetobacter lwoffii*	(90)
6.12 g/d of RPM (10 weeks)	↑*Succiniclasticum*, ↑*Selenomonas*, ↑*Clostridium_XlVa*	(91)
L-Met 0.28% (in vitro culture)	↑*Campilobacterota*, ↑*Proteobacteria*; ↓*Verrucomicrobiota*	(92)
80 mg Zn-Met/d (2 weeks)	↑*Bacteroides*, ↑*Lactobacillus*, ↑*Faecalibacterium*; ↓*Proteobacteria*	(93)
Met 0.09% of DMI (28 days prepartum to 42 days postpartum)	↑*Ruminococcus Dialister*, ↑*Fusobacterium,* ↑*Fusobacteriaceae,* ↑*Fusobacteriales;* ↓*Actinomyces,* ↓*Bifidobacterium* ↓*Bifidobacteriales,* ↓*Prevotella and Burkholderiales*	(25)

#### 4.1.1 Enhancement of beneficial microbial populations

As indicated in Table 1, supplementation with RPM leads to a marked increase in the abundance of *Eubacterium ruminantium* and *Selenomonas ruminantium*, with the latter’s abundance nearly doubling. *Selenomonas ruminantium*, a core symbiotic bacterium in the rumen microbiome, can comprise up to 51% of the total viable microbial population in the rumen of ruminants (23, 24). This increase in microbial abundance is associated with improved energy metabolism within the intestinal epithelium, enhanced gut motility, improved nutrient absorption, and the prevention of gastrointestinal disturbances such as diarrhea. Additionally, *Ruminococcus Dialister* and *Ruminococcus albus*, which are involved in the degradation of phytates and cellulose, respectively, play critical roles in improving intestinal barrier integrity and mitigating inflammation (25). Met supplementation also exerts a significant modulatory effect on the growth of beneficial intestinal bacteria, including *Lactobacillus* species (LAB) and *Bifidobacterium*. LAB, through the fermentation of carbohydrates, produces lactic acid, which lowers intestinal pH and inhibits the growth of pathogenic microbes. Furthermore, LAB have demonstrated anti-inflammatory effects and contribute to the maintenance of gut barrier integrity while modulating immune responses, thereby enhancing pathogen resistance, particularly during the periparturient period in dairy cows (26–28). Specific strains of *Bifidobacterium*, such as *Bifidobacterium breve* M1 and M2, have been shown to alleviate inflammation in DSS-induced colitis by producing conjugated linoleic acid, suppressing pro-inflammatory cytokines, and preserving intestinal epithelial integrity (29–33).

#### 4.1.2 Inhibition of pathogenic bacterial proliferation

Enterotoxigenic *Escherichia coli* (ETEC), a pathogenic strain of *E. coli*, is a major causative agent of watery diarrhea in neonatal calves (34). Similarly, Salmonella, a Gram-negative bacterium from the Enterobacteriaceae family, is recognized as a significant pathogen in cattle, leading to systemic illnesses such as enteritis, septicemia, and abortion. Salmonella strains exhibit resistance to various antibiotics, including tetracycline, ampicillin, and sulfamethoxazole, complicating treatment strategies (35). Infections with Salmonella in dairy cattle contribute to severe clinical syndromes, including gastroenteritis and systemic infections (36). Met has demonstrated substantial inhibitory effects on the proliferation of harmful intestinal pathogens, including *E. coli* and *Salmonella typhimurium*, in other animal species (37). By modulating the gut microbial composition, met supplementation helps optimize microbial balance and enhance gut resilience against pathogenic invasion. This effect is of considerable practical significance in dairy farming, as the strategic inclusion of Met in the diet can help improve intestinal microbial ecology, bolster the gut’s defensive capabilities, and thereby elevate overall animal health. The antimicrobial properties of met make it a key component in enhancing gut health and protecting dairy cattle from enteric infections (37).

### 4.2 Chemical barrier in the intestine

Short-chain fatty acids (SCFAs), which are produced by microbial fermentation of dietary fiber, play a central role in regulating the expression of mucin (MUC) genes at the transcriptional level. SCFAs, including acetate, propionate, and butyrate, enhance the synthesis and secretion of mucins, which are essential components of the intestinal chemical barrier. The mucous layer, primarily composed of MUC proteins, acts as a critical defense mechanism by physically blocking the entry of pathogens and limiting damage to the intestinal epithelium. Moreover, SCFAs contribute to maintaining intestinal homeostasis by enhancing mucosal function and promoting epithelial cell turnover (38). Met influences the metabolic pathways within intestinal epithelial cells by donating methyl groups through its conversion to S-adenosylmethionine (SAM). SAM, a critical methyl donor, supports the methylation of homocysteine (Hcy) and facilitates the biosynthesis of essential metabolites such as cysteine and GSH. These metabolites are integral for maintaining cellular redox balance, supporting antioxidant defenses, and promoting immune function. Through methylation, SAM contributes to the glycosylation and post-translational modification of mucins, ensuring the integrity and function of the intestinal mucosal layer. Additionally, Met metabolism provides cysteine, a key precursor for mucin synthesis, which is vital for the repair and maintenance of the mucosal barrier (38). Furthermore, dietary supplementation with Zn-Met has been shown to enhance the expression of MUC2 and other mucin genes, thereby strengthening the intestinal chemical barrier. The increased synthesis of mucins via Zn-Met supplementation not only improves the mechanical barrier but also reinforces the chemical defense by promoting the production of antimicrobial peptides and enhancing gut resilience to pathogen invasion (39, 40). These findings emphasize the critical role of Met and its derivatives in modulating both microbial and chemical barriers to maintain intestinal health and function (Tables 2–5).

**Table 2:** Summary of the effects of met supplementation on the chemical barrier function in the gastrointestinal tract of dairy cows.

Supplementation level and duration	Observed effects	Reference
Met at 0.09% of DM (28 days prepartum to 42 days postpartum)	↑Short-chain fatty acid (SCFA) production	(25)
6.12 g/d of RPM Dipeptide (10 weeks)	↑Acetate, and valerate concentrations in the rumen	(91)
6 g/d of DL-Met (5 weeks)	↑Acetic acid but no effect on other SCFA	(94)
455 mg Zn-Met/d (2 weeks)	↑Propionate, butyrate, and isovalerate; promoted mucin synthesis	(90)

**Table 3:** Summary of studies investigating the effects of met and its derivatives on intestinal mechanical barrier function in holstein dairy cows.

Maternal supplementation level and duration	Effects on intestinal barrier function	Reference
455 mg Zn-Met/d (2 weeks)	↑*ACOX1*, *FABP2*, and *PPAR-γ* in the jejunal mucosa; ↑Lipid metabolism; ↑mechanical barrier integrity	(90)
455 mg Zn-Met/d (2 weeks)	↑Ileal villus height; ↑tight junction proteins *claudin-1*, *occludin*, and *ZO-1;* ↓gut permeability	(48)
0.9 g RPM/kg DMI(4 weeks)	↑Glucose and amino acid transporter protein in the jejunal mucosa	(57)
80 mg Zn-Met/d (2 weeks)	↓Diarrhea incidence	(93)
455 mg Zn-Met/d (2 weeks)	↑Zinc transporter protein *ZIP4* in the jejunal mucosa	(95)

**Table 4:** Summary of studies investigating the effects of met and its derivatives on immune function in holstein dairy cows.

Supplementation level and duration	Comprehensive immunological effects	Reference
60 mg/kg DM of Zn-Met (60 days until parturition)	↑IL-2 and IL-6; ↑B cell proliferation and differentiation	(96)
RPM at 0.09 and 0.10% of DMI (28 days prepartum to 30 days postpartum)	↑*GPX1*, *GPX3*, *GSTM1*, and *GSTA4*; ↑GSH metabolism and the antioxidant NFE2L2 signaling pathway	(97)
RPM at 0.09 and 0.10% of DMI (28 days prepartum to 60 days postpartum)	↑PMN oxidative burst and phagocytic activity; ↓oxidative stress and inflammation	(82)
RPM at 0.08% of DM (21 days prepartum to 30 days postpartum)	↑redox function of polymorphonuclear leukocytes (PMNLs); ↓inflammation and oxidative stress	(98)
RPM at 0.08% of DM (21 days prepartum to 30 days postpartum)	↑PMNL and monocyte phagocytic capacity; ↑oxidative burst activity	(22)
RPM at 0.08% of DM, Lys: Met ratio 2.8:1 (21 days prepartum to 73 days postpartum)	↓*IL-1β*, *IL-6*, and *IL-8*; ↑immune homeostasis	(99, 100)
RPM at 0.09% of DM (28 days prepartum to 50 days postpartum)	↑PMN phagocytic activity; ↑oxidative burst capacity	(101)

**Table 5:** Summary of studies investigating the effects of met with or without lysine supplementation on lactation performance in dairy cows.

Supplementation level and duration	Observed effects	Reference
RPL 100 g/day and RPM 30 g/day (8 weeks)	↑Milk yield under a low-protein diet (14.2% CP)	(102)
RPL 110 g/day and RPM 31 g/day (8 weeks)	↑Milk yield; ↑milk protein yield in cows fed a 15% CP diet	(103)
RPL 60 g/day and RPM 25 g/day (3 weeks)	Maintained milk yield and composition under a severely MP-deficient diet	(104)
Met 16.5 g/day (11 weeks)	↑Milk protein yield in spring-calving cows	(105)
0.17% RPM and 0.41% RPL of DM (22 to 150 DIM)	↑Postpartum DMI, milk yield, and milk protein production	(106)
Rumen-protected Met+Lys (RPML) 107 g/day (6 weeks)	↑Milk yield in mid-lactation, without altering milk composition	(107)
RPM (Smartamine M, 9 g/day Met) (3 weeks)	↑Milk protein concentration	(108)
Met 30 g/day (NALM) (13 weeks)	↑Milk yield; ↓lipid peroxidation during mid-lactation	(109)

Antimicrobial peptides (AMPs), such as defensins and cathelicidins, are secreted by Paneth cells, and exhibit broad-spectrum antimicrobial activity against antibiotic-resistant bacteria. These peptides disrupt the integrity of bacterial cell membranes, leading to bactericidal effects (41). The permeabilization of microbial cell membranes is a hallmark of the antimicrobial activity mediated by defensins. In the bovine small intestine and colon, *β*-defensins are electrostatically adsorbed to the anionic sites on target cell membranes, promoting the aggregation and polymerization of defensins. Under the influence of the membrane potential, the positively charged defensins insert into the lipid bilayer of the cell membrane, disrupting osmotic pressure and ionic balance, ultimately leading to bacterial cell death (42). Studies on defensin-mediated antimicrobial activity have demonstrated that after pathogenic infection, the concentration of defensins in the intestinal mucosa increases, contributing to antibacterial responses (43, 44). Mechanistic target of rapamycin (mTOR), a key nutrient-sensing factor, is a critical regulator of immune responses (33). When S-adenosylmethionine (SAM), produced through Met metabolism, binds to SAMTOR (S-adenosylmethionine sensor for mTOR), it inhibits the interaction between SAMTOR and GATOR1, thereby activating mTOR complex 1 (mTORC1). This activation enhances cell proliferation and the production of defensins (45). Although some studies investigate mechanisms in humans, similar pathways may potentially occur in cattle; however, direct evidence in ruminants is currently limited. Therefore, the extrapolation of these mechanisms to dairy cows should be interpreted cautiously and requires further investigation.

### 4.3 Strengthening of the intestinal mechanical barrier

In randomized trials, the use of Zn-Met supplementation was compared to zinc oxide (ZnO) supplementation and a control group (CON) to evaluate its effects on intestinal permeability and epithelial barrier integrity in dairy calves. The results demonstrated that Zn-Met supplementation significantly reduced intestinal permeability and improved the integrity of the intestinal epithelial barrier (46–48). Zn-Met supplementation promoted the proliferation and differentiation of intestinal mucosal cells, providing a sufficient cellular source for the growth of intestinal villi, thereby increasing the surface area available for nutrient absorption. Key parameters such as villus height (V), crypt depth (C), and the villus-to-crypt ratio (V/C) are critical for optimizing nutrient absorption in dairy cattle (49, 50). Notably, supplementation with Zn-Met, but not ZnO, exerted a significant effect on the morphology of the small intestine in postpartum Holstein calves. However, the villus height, crypt depth, and V/C ratio of the duodenum and jejunum were unaffected by either Zn-Met or ZnO supplementation (48).

Tight junction (TJ) proteins, including claudins, occludin, and zonula occludens (ZO) proteins, play a central role in maintaining the intestinal epithelial barrier. These proteins are linked to the actin cytoskeleton through cytoplasmic plaque proteins such as TJP1/ZO-1, TJP2/ZO-2, TJP3/ZO-3 (51). Disruption of the TJ barrier, accompanied by a reduction in the expression of TJ proteins, results in increased intestinal permeability, allowing the translocation of microorganisms and harmful water-soluble molecules into the epithelial cells, which can lead to inflammatory bowel disease (IBD) (52–54). Studies have shown that Met supplementation promotes the expression of intestinal TJ proteins by activating the JAK2/STAT3 signaling pathway (55). Furthermore, DL-Met has been shown to activate the Wnt/*β*-catenin signaling pathway, which controls cell proliferation and differentiation, thereby enhancing the expression of TJ proteins (56). Supplementation with Zn-Met significantly increased the transcriptional levels of claudin-1, occludin, and ZO-1 in the jejunal mucosa, subsequently reducing intestinal permeability and decreasing the incidence of diarrhea in postpartum dairy cows (48, 57). These findings underscore the critical role of Met and its derivatives, such as Zn-Met, in enhancing intestinal barrier function and overall gut health in dairy cattle.

### 4.4 Immune function

The immune system is a pivotal component of the bovine immune response, playing a crucial role in defending against pathogen invasion and maintaining intestinal microbial homeostasis. Antimicrobial peptides (AMPs), such as defensins and cathelicidins, are key effectors within the immune barrier, exerting broad-spectrum antimicrobial activity. Upon pathogen challenge, the NF-κB signaling pathway is activated, leading to an upregulation of pro-inflammatory cytokines (e.g., IL-1β, TNF-*α*, and IL-8), thereby enhancing the host immune response (58). In parallel, the mitogen-activated protein kinase (MAPK) pathway is implicated in the regulation of cytokine gene expression and subsequent protein secretion, thereby contributing to the systemic inflammatory response. Evidence suggests that cathelicidin-BF modulates macrophage phagocytic activity through the activation of STAT-1 signaling, which in turn facilitates the production and secretion of interleukins (IL) and interferons (IFN), thus orchestrating immune modulation (59).

Met serves as a critical modulator of the immune functions in dairy cattle, principally through its influence on immune cell activation and the secretion of immune mediators. The immune system can be classified into innate and adaptive components, which are interlinked through cytokine signaling pathways. Adaptive immunity is characterized by humoral factors and the activation of T and B lymphocytes, whereas the innate immune response involves cellular players such as macrophages, dendritic cells, and polymorphonuclear leukocytes (PMNs) (60, 61). Macrophages, during the process of pathogen phagocytosis, generate cytokines, including IFN-*γ*, IL-6, and TNF-*α*, which activate both innate and adaptive immune pathways. This response enhances the recruitment of PMNs to the site of infection, while also promoting pro-inflammatory and antimicrobial activities essential for pathogen clearance (62, 63).

Met, via its metabolic pathway, is a primary contributor to the synthesis of GSH, the predominant endogenous antioxidant in the body, known for its capacity to neutralize free radicals and reactive oxygen species (ROS). Supplementation with Met has been shown to enhance T lymphocyte proliferation and improve the bactericidal activity of neutrophils, thus potentiating the immune response through augmented oxidative burst capacity. Furthermore, the administration of RPM during mid-lactation (28 days) has been reported to significantly enhance the proliferative capacity of peripheral blood T lymphocytes, thereby bolstering the adaptive immune response (64). Additionally, Met supplementation has been linked to reduced brain phospholipid concentrations and a concomitant reduction in the synthesis of pro-inflammatory cytokines, such as IL-6, thereby alleviating inflammatory responses. Metabolic byproducts of Met, including taurine, GSH, and homocysteine (Hcy), are integral to enhancing immune modulation in dairy cows. Supplementation with RPM has been shown to elevate total antioxidant capacity, glutathione peroxidase (GPX) activity, and vitamin E levels, which are recognized biomarkers of oxidative stress amelioration (33, 65). Moreover, sufficient Met availability has been demonstrated to significantly augment the growth and cellulolytic activity of rumen microorganisms, further enhancing gut health and microbial balance (66). To date, direct evidence linking methionine supplementation to intestinal immune function in dairy cattle is limited. The precise mechanism by which methionine may influence intestinal outcomes in dairy cows remains unclear. Methionine is a key methyl donor and contributes to antioxidant defense, protein synthesis, and cellular signaling, which could potentially affect gut immune function. However, direct mechanistic studies in ruminants are limited, and further research is required to elucidate these pathways.

## 5 Impact of met on milk production and reproductive performance

### 5.1 Effects on milk production performance

Extensive research has investigated the effects of RPM supplementation on milk production. While several individual studies have reported improvements in milk yield as well as milk fat and protein concentrations (67–70), a recent meta-analysis indicated that the most consistent benefits of RPM supplementation are observed in milk protein and fat percentages, with limited or inconsistent effects on overall milk yield when compared with a basal diet (71). These findings suggest that the primary advantage of RPM lies in improving milk composition rather than markedly increasing milk volume. More recently, a systematic review and meta-analysis by Zanton and Toledo (72) reported that RPM supplementation before and after calving was associated with increased milk yield, particularly during early lactation. Taken together, these findings suggest that while meta-analytic evidence has varied depending on study selection and methodological approaches, the overall consensus supports a consistent benefit of RPM for milk composition, with potential yield responses under specific physiological conditions such as the transition period. Nonetheless, in practical applications, particularly during the periparturient period, the inclusion of RPM in combination with rumen-protected lysine (RPL) in the diet has been shown to more effectively enhance milk yield than the provision of single amino acids (72, 73). This suggests that the synergistic effects of multiple amino acids may optimize dairy cattle performance beyond the effects of a single AA supplementation. However, the optimal dose, timing, and duration of Met supplementation to maximize lactation performance remain to be clearly defined.

### 5.2 Impact on reproductive performance

#### 5.2.1 Influence on pregnancy rates and embryonic development

Met plays an essential role in the formation of embryonic tissues and organs during gestation, with early embryonic development often being closely associated with Met concentrations (74–76). Numerous studies have demonstrated that supplementation with RPM can effectively reduce pregnancy loss and improve embryonic size in multiparous dairy cows over specific periods of gestation (77). Furthermore, reproductive trait analysis, utilizing pregnancy-specific protein B (PSPB) concentrations, has substantiated that RPM supplementation significantly mitigates the incidence of pregnancy loss during critical stages of gestation. RPM supplementation has also been shown to enhance lipid deposition in embryos, providing a crucial energy source for early-stage embryonic development. This process contributes to improved embryonic viability and quality, thereby reducing the risk of miscarriage and congenital anomalies (76). However, it is noteworthy that recent studies found that periparturient supplementation with MHA does not yield significant improvements in offspring growth or final pregnancy rates (78, 79). In addition, Peñagaricano et al. (80) demonstrated that maternal methionine supplementation during early reproductive stages can alter the transcriptome of bovine preimplantation embryos. Their study showed that approximately 72% of differentially expressed genes were downregulated, which the authors suggested may result from increased DNA methylation due to higher levels of methyl donors, potentially suppressing fetal gene expression. These findings highlight that while methionine can positively influence reproductive performance, its role in early embryonic development may involve complex epigenetic regulation that warrants further investigation. In this regard, the individual variability in response to Met supplementation—potentially due to genetic background, diet composition, health status, or environmental conditions—needs to be characterized in the future.

#### 5.2.2 Influence on periparturient health

Periparturient health problems in dairy cows refer to the physiological and metabolic disorders that commonly occur during the transition period, which spans from three weeks before to three weeks after calving. This is a critical time, as cows undergo significant hormonal, metabolic, and nutritional changes to support calving and the onset of lactation. Health problems during this period can severely impact milk production, reproduction, and overall animal welfare. RPM supplementation significantly alleviates oxidative stress associated with parturition, mitigating exaggerated inflammatory responses and improving the periparturient immune-metabolic function of dairy cows (81). Furthermore, RPM supplementation reduces the oxidative stress associated with iron metabolism and lowers plasma levels of reactive oxygen metabolites (ROMs). Concurrently, it increases plasma concentrations of key antioxidant biomarkers, including *β*-carotene, tocopherol, total glutathione, and reduced GSH, thereby bolstering the antioxidant defense system (82).

Studies suggest that early postpartum Met supplementation benefits Holstein cows affected by bacterial endometritis (83). Additionally, polymorphonuclear neutrophil (PMN) function is associated with uterine infections, including retained placenta (RP), endometritis, and uterine infections. Experimental evidence indicates that Met supplementation enhances PMN phagocytic and oxidative burst activity in dairy cows following pathogen exposure, thus improving uterine inflammation outcomes (22). Recent studies have demonstrated that supplementing RPM in the diet 21 days prior to calving and 30 days postpartum effectively enhances hepatic GSH levels, improves liver function, and modulates plasma biomarkers of inflammation. These changes contribute to increased neutrophil phagocytic activity and oxidative burst capacity (9, 22, 33).

Dairy cows in the periparturient period often experience a state of negative energy balance. Met participates in the one-carbon metabolic pathway, providing methyl groups via methylation reactions that facilitate the synthesis of SAM. SAM serves as a critical methyl donor for purine and pyrimidine synthesis, thereby supporting cellular energy metabolism and gene expression (31, 32). Additionally, Met is heavily involved in the sulfur cycle, where it is enzymatically converted into L-Met sulfoxide, playing an essential role in protein translation, GSH synthesis, and taurine production (7, 84, 85). Research investigating the individual responses of dairy cattle to RPM supplementation has revealed that it significantly enhances AA metabolism. Key indicators include increased mammary blood flow (MBF), elevated mammary gland AA uptake and clearance rates, and improved AA uptake-to-output ratios, all of which provide evidence of enhanced metabolic efficiency in lactating cows (86). Moreover, during the periparturient period, increased demands for milk protein synthesis and gluconeogenesis drive enhanced amino acid recycling, with experimental findings showing elevated plasma concentrations of multiple amino acids (Ala, Arg, Lys) and their derivatives, such as cysteine, in Met-supplemented dairy cows (87–89). Met also contributes to phospholipid synthesis, facilitating the transport of triglycerides from the liver in lipoprotein form to peripheral tissues, thereby reducing hepatic fat accumulation.

## 6 Conclusion

This review offers a comprehensive evaluation of the roles of Met and its derivatives in dairy cow nutrition, with particular emphasis on their beneficial effects on intestinal barrier function, periparturient physiological performance, and systemic metabolic health. Accumulating evidence underscores the potential of Met, particularly in its rumen-protected form, as a critical dietary component for enhancing gut integrity, modulating immune responses, and supporting reproductive function in high-producing dairy cattle. Despite these advancements, several important knowledge gaps persist.

First, the precise molecular and cellular mechanisms by which Met influences intestinal and reproductive physiology remain incompletely understood, particularly in the context of immune signaling pathways, epithelial restitution, and endocrine regulation. Second, the interactions between dietary Met and the gut microbiota—including changes in microbial composition, metabolite production, and their subsequent effects on host nutrient absorption and barrier function—warrant further investigation. Third, the optimal dose, timing, and duration of Met supplementation to maximize physiological benefits remain to be clearly defined, particularly during the transition period. Fourth, individual variability in response to Met supplementation—potentially due to genetic background, diet composition, health status, or environmental conditions—is not well characterized. Finally, the long-term impacts of sustained RPM use on animal health, reproductive lifespan, and productive efficiency across multiple lactations remain largely unexplored.

To address these gaps, future research should employ integrative multi-omics approaches (e.g., transcriptomics, proteomics, metabolomics) to elucidate the mechanistic underpinnings of Met action in gut and reproductive tissues. Concurrently, microbiota-metabolite profiling may provide insight into host-microbiome interactions mediated by Met. The development of precision nutrition models that incorporate real-time physiological and metabolic data will allow for individualized supplementation strategies. Furthermore, exploring synergistic effects of Met with other functional nutrients (e.g., choline, lysine, trace elements) may yield novel strategies for enhancing gastrointestinal and reproductive function. Finally, translational research conducted under commercial dairy production conditions is essential to validate experimental findings and support the development of evidence-based dietary guidelines for methionine supplementation. Collectively, these research directions will advance the understanding and application of Met as a functional nutrient in dairy cow nutrition.
